# Incidence, Risk Factors and Impact on Long-Term Outcome of Postoperative Delirium After Transcatheter Aortic Valve Replacement

**DOI:** 10.3389/fcvm.2021.645724

**Published:** 2021-03-26

**Authors:** Victor Mauri, Kevin Reuter, Maria I. Körber, Hendrik Wienemann, Samuel Lee, Kaveh Eghbalzadeh, Elmar Kuhn, Stephan Baldus, Malte Kelm, Georg Nickenig, Verena Veulemans, Felix Jansen, Matti Adam, Tanja K. Rudolph

**Affiliations:** ^1^Department of Cardiology, Faculty of Medicine, Heart Center, University of Cologne, Cologne, Germany; ^2^Department of Cardiothoracic Surgery, Faculty of Medicine, Heart Centre, University of Cologne, Cologne, Germany; ^3^Division of Cardiology, Pulmonology and Vascular Medicine, Medical Faculty, Heinrich Heine University, Düsseldorf, Germany; ^4^CARID (Cardiovascular Research Institute Düsseldorf), Medical Faculty, Heinrich Heine University, Düsseldorf, Germany; ^5^Department of Medicine II, Heart Center Bonn, University Hospital Bonn, Bonn, Germany; ^6^General and Interventional Cardiology, Heart and Diabetes Centre Nordrhein-Westfalen, Bad Oeynhausen, Germany; ^7^Medical Faculty, Ruhr University Bochum, Bochum, Germany

**Keywords:** delirium, frailty, survival, TAVR, transcatheter aortic valve implantation

## Abstract

**Background:** The aim of the present study was to analyze incidence, risk factors, and association with long-term outcome of postoperative delirium (POD) after transcatheter aortic valve replacement (TAVR).

**Methods:** Six hundred and sixty one consecutive patients undergoing TAVR were prospectively enrolled from January 2016 to December 2017. POD was assessed regularly during ICU-stay using the CAM-ICU test.

**Results:** The incidence of POD was 10.0% (*n* = 66). Patients developing POD were predominantly male (65%), had higher EuroSCORE II (5.4% vs. 3.9%; *P* = 0.041) and were more often considered frail (70% vs. 26%; *P* < 0.001). POD was associated with more peri-procedural complications including vascular complications (19.7 vs. 9.4; *P* = 0.017), bleeding (12.1 vs. 5.4%; *P* = 0.0495); stroke (4.5 vs. 0.7%; *P* = 0.025), respiratory failure requiring ventilation (16.7% vs. 1.8%; *P* < 0.001), and pneumonia (34.8% vs. 7.1%; *P* < 0.001). Consequently, patients with POD had significantly longer ICU- (7.9 vs. 3.2 days *P* < 0.001) and hospital-stay (14.9 vs. 9.0 days; *P* < 0.001), and higher in-hospital mortality (6.1 vs. 2.1%; *P* = 0.017). Logistic regression analysis identified male sex (odds ratio (OR) 2.2 [95% confidence interval (CI) 1.2–4.0); *P* = 0.012], atrial fibrillation [OR 3.0 (CI 1.6–5.6); *P* < 0.001], frailty [OR 4.3 (CI 2.4–7.9); *P* < 0.001], pneumonia [OR 4.4 (CI 2.3–8.7); *P* < 0.001], stroke [OR 7.0 (CI 1.2–41.6); *P* = 0.031], vascular complication [OR 2.9 (CI 1.3–6.3); *P* = 0.007], and general anesthesia [OR 2.0 (CI 1.0–3.7); *P* = 0.039] as independent predictors of POD. On Cox proportional hazard analysis POD emerged as a significant predictor of 2-year mortality [HR 1.89 (CI 1.06–3.36); *P* = 0.030].

**Conclusion:** POD is a frequent finding after TAVR and is significantly associated with reduced 2-year survival. Predictors of delirium include not only peri-procedural parameters like stroke, pneumonia, vascular complications and general anesthesia but also baseline characteristics as male sex, atrial fibrillation and frailty.

## Introduction

Postoperative delirium (POD) is a common organic brain syndrome characterized by an acute onset of neurocognitive dysfunction (1). POD is a frequent finding after cardiac surgery. Incidence is increasing with age affecting up to 55% of patients aged ≥ 70-years (2). Although mostly transient, POD has been associated with prolonged hospital stay, long-lasting functional and cognitive decline, substantially increased health care costs, and higher perioperative and long-term mortality (3–9). Since it is potentially preventable, POD may be an important target for supportive interventions to improve patient outcome (10). Transcatheter aortic valve replacement (TAVR) has emerged as clinical standard for the treatment of severe aortic stenosis in elderly patients and patients considered at increased risk for conventional surgery (11). Characterized by advanced age, frailty, and multiple comorbidities, patients undergoing TAVR seem at particular high risk to develop POD. However, the lack of cardiopulmonary bypass, conscious sedation and early mobilization may lower the risk of POD compared to surgery. Data on the incidence and especially long-term consequences of delirium after TAVR are scarce. With the present study, we sought to investigate incidence and risk factors of POD after TAVR, as well as whether the occurrence of POD would have a negative impact on long-term survival.

## Methods

This study includes all 661 consecutive patients undergoing TAVR for severe native aortic stenosis between January 2016 and December 2017 at Cologne University Heart Center. All patients were evaluated by an interdisciplinary heart-team and percutaneous therapy was chosen based on individual surgical risk and patient characteristics following current guidelines (11). The study was approved by the institutional ethics committee (19-1032). Baseline demographic and clinical characteristics were retrieved from electronic medical records and entered in a dedicated database. Frailty was assessed with the Essential Frailty Toolset (EFT) as described previously (12). In brief, the EFT is scored 0 (least frail) to 5 (most frail) based on the four items pre-procedural anemia, hypoalbuminemia, lower-extremity muscle weakness and cognitive impairment. Clinical and safety endpoints are reported according to the VARC-2 consensus (13). Major adverse event (MAE) was defined as a composite of stroke, major vascular complication, major or life-threatening bleeding, and stage 2 or 3 acute kidney injury.

### Assessment of POD

POD was assessed with a two-step approach following current recommendations (14): After evaluation of sedation and arousal with the Richmond Agitation Sedation Scale (RASS) (15), the Confusion Assessment Method for the Intensive Care Unit (CAM-ICU) was used in case of a RASS score of −3 or higher. In the case of RASS score of −4 or −5 (comatose state without reaction to verbal stimulation), POD was reassessed at a later time point. The CAM-ICU is a validated tool to assess delirium based on the four principle features of delirium derived from the Diagnostic and Statistical Manual of Mental Disorders: Acute onset or fluctuating course of mental status change (1), inattention (2), disorganized thinking (3), and altered level of consciousness (4). Delirium is defined as combination of both features (1) and (2) plus either feature (3) or (4) (1, 16). POD was assessed on the first and second postoperative day for every patient by trained staff, and additional assessment up to 7 days after the initial procedure in case of suspected delirium by the treating nurse or attending physician. Delirium was considered present if at least 1 CAM-ICU assessment was positive during the study period. Early mobilization after TAVR was encouraged as preventive measure and medical treatment was used according to the discretion of the treating physician.

### Statistical Analysis

Continuous variables are presented as mean ± standard deviation, while categorical variables are reported as frequencies and percentages. Differences between patients with and without POD were evaluated using Fisher's exact test for categorical variables and Student's *t*-test or Mann-Whitney-U test for continuous variables, depending on their distribution. Logistic regression analysis was used to identify independent predictors of POD. Kaplan-Meier curves were drawn to estimate 2-year survival and compared using the log-rank test. Cox proportional hazards model was used to adjust for confounders of mortality including age, sex, EuroSCORE II, chronic kidney disease, atrial fibrillation, frailty, and major peri-procedural complications Two-sided *P* < 0.05 were considered statistically significant. All statistical analyses were performed with IBM SPSS Statistics, Version 25.

## Results

Six hundred and sixty one patients underwent TAVR for native severe aortic stenosis during the study period and were included into the analysis. Mean age was 82 ± 6-years, mean EuroSCORE II was 4.0 ± 3.6%, and 51% were female (Table 1). Frequent comorbidities include hypertension (88%), coronary artery disease (63%), atrial fibrillation (43%), and chronic kidney disease (GFR 54 ± 21 ml/min; 60%). Thirty percentage of patients were considered frail (EFT Score ≥ 3).

**Table 1:** Baseline and procedural characteristics of patients with and without postoperative delirium.

**Parameter**	**All (*n* = 661)**	**Delirium (*n* = 66)**	**No Delirium (*n* = 595)**	***P***
**Baseline parameters**				
Age	82.3 ± 6.6	82.4 ± 5.1	82.2 ± 5.7	0.904
Male sex	322 (48.7)	43 (65.2)	279 (46.9)	0.006
Diabetes Mellitus	208 (31.4)	16 (24.2)	192 (32.3)	0.210
Hypertension	581 (87.9)	62 (93.9)	519 (87.2)	0.161
Atrial fibrillation	282 (42.6)	45 (68.2)	237 (39.8)	<0.001
Renal disease	398 (60.2)	46 (69.7)	352 (59.2)	0.112
Chronic lung disease	111 (16.7)	13 (19.7)	98 (16.5)	0.490
Peripheral artery disease	132 (20.0)	20 (30.3)	112 (18.8)	0.034
Coronary artery disease	415 (62.8)	47 (71.2)	368 (61.8)	0.142
Previous cardiac surgery	71 (10.7)	10 (15.2)	61 (10.3)	0.213
Frailty (EFT ≥ 3)	199 (30.1)	46 (69.7)	153 (25.7)	<0.001
EuroSCORE II	4.0 ± 3.6	5.4 ± 5.4	3.9 ± 3.3	0.041
**Procedural aspects and complications**				
General anesthesia	373 (56.4)	48 (72.7)	325 (54.6)	0.006
Non-transfemoral access	17 (2.6)	3 (4.5)	14 (2.4)	0.236
Vascular complications	69 (10.4)	13 (19.7)	56 (9.4)	0.017
Bleeding	40 (6.1)	8 (12.1)	32 (5.4)	0.0495
Stroke	7 (1.1)	3 (4.5)	4 (0.7)	0.025
Respiratory failure requiring ventilation	22 (3.3)	11 (16.7)	11 (1.8)	<0.001
Stage 2/3 acute kidney injury	20 (3.0)	4 (6.1)	16 (2.7)	0.129
Pneumonia	65 (9.8)	23 (34.8)	42 (7.1)	<0.001
Antibiotic treatment	119 (18.0)	30 (45.5)	89 (15.0)	<0.001
Need for blood transfusion	77 (11.6)	17 (25.8)	60 (10.1)	0.001
Major adverse event	46 (7.0)	10 (15.2)	36 (6.1)	0.017
In-hospital mortality	11 (1.7)	4 (6.1)	7 (1.2)	0.017

*Values are mean ± standard deviation or n (%)*.

The incidence of POD was 10% (*n* = 66). Baseline characteristics of patients with and without POD are shown in Table 1. Patients developing POD were predominantly male (65%; *P* = 0.006), had higher EuroSCORE II (5.4% vs. 3.9%; *P* = 0.041) and were more often considered frail (70 vs. 26%; *P* < 0.001). The prevalence of atrial fibrillation (68 vs. 40%; *P* < 0.001) was significantly higher in POD patients. Patients with delirium had undergone TAVR more often under general anesthesia instead of conscious sedation (73 vs. 54%; *P* = 0.006).

Postoperative delirium was associated with a higher rate of peri-procedural complications including vascular complications (all 19.7 vs. 9.4%, *P* = 0.017; major 7.6 vs. 2.5%, *P* = 0.040), bleeding (all bleeding, 12.1 vs. 5.4%, *P* = 0.0495; major/life-threatening 6.1 vs 1.8%, *P* = 0.053), stroke (4.5 vs. 0.7%; *P* = 0.025), respiratory failure requiring prolonged mechanical ventilation (16.7% vs. 1.8%; *P* < 0.001), pneumonia (34.8% vs. 7.1%; *P* < 0.001), and need for antibiotic treatment (45.5 vs. 15.0%; *P* < 0.001). Patients who developed POD more often required blood transfusions (25.8% vs. 10.1%, *P* = 0.001). Out of the 66 patients with POD, 41 cases (62.1%) were associated with one of those complications. Consequently, patients with POD had significantly longer ICU- (7.9 vs. 3.2 days *P* < 0.001) and hospital-stay (14.9 vs. 9.5 days; *P* < 0.001).

On multivariable logistic regression analysis, male sex [odds ratio (OR) 2.2 (95% confidence interval (CI) 1.2–4.0); *P* = 0.012], atrial fibrillation [OR 3.0 (CI 1.6–5.6); *P* < 0.001], frailty [OR 4.3 (CI 2.4–7.9); *P* < 0.001], pneumonia [OR 4.4 (CI 2.3–8.7); *P* < 0.001], stroke [OR 7.0 (CI 1.2–41.6); *P* = 0.031], vascular complication [OR 2.9 (CI 1.3–6.3); *P* = 0.007], and general anesthesia [OR 2.0 (CI 1.0–3.7); *P* = 0.039] emerged as independent predictors of POD (Table 2).

**Table 2:** Predictors of postoperative delirium after TAVR.

**Parameter**	**Odds ratio**	**95%-confidence interval**	***P***
Male sex	2.18	1.19–3.98	0.012
General anesthesia	1.96	1.04–3.72	0.039
Atrial fibrillation	3.04	1.63–5.65	<0.001
Vascular complication	2.9	1.35–6.30	0.007
Pneumonia	4.44	2.26–8.72	<0.001
Frailty (EFT ≥ 3)	4.31	2.37–7.87	<0.001
Stroke	7.03	1.19–41.58	0.031

**Figure 1:**
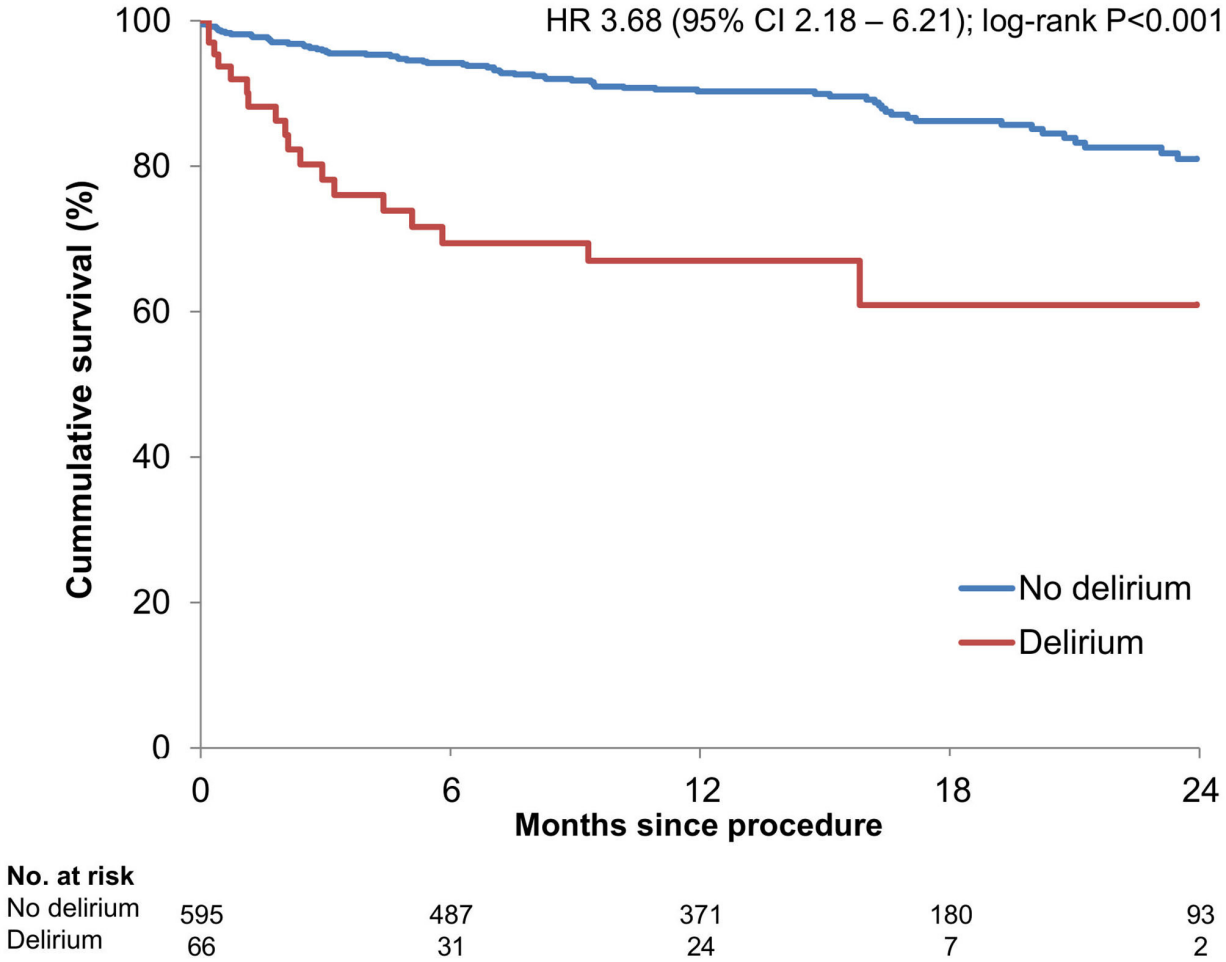
Kaplan Meier survival estimates stratified by postoperative delirium.

Median follow-up time was 384 days (IQR 226–518), total follow up time was 689 patient years. In-hospital mortality (6.1 vs. 1.2%; *P* = 0.017) and estimated 2-year mortality [39.1 vs. 19.0%; HR 3.68 (95% CI 2.18–6.21); log-rank *P* < 0.001] were significantly higher in patients with POD (Figure 1). After adjustment for age, sex, EuroSCORE II, chronic kidney disease, atrial fibrillation, frailty, and major peri-procedural complications, only relevant peri-procedural complications remained significantly associated with in-hospital mortality, whereas POD did not. However, on multivariable Cox proportional hazards analysis POD remained a significant predictor of 2-year mortality [HR 1.9 (CI 1.1–3.4); *P* = 0.030], even after adjustment for age, sex, EuroSCORE II, chronic kidney disease, atrial fibrillation, frailty, and major peri-procedural complications (Table 3).

**Table 3:** Predictors of 2-year mortality.

**Parameter**	**Hazard ratio**	**95%-confidence interval**	***P***
Age	1.00	0.96–1.04	0.912
Male sex	2.04	1.29–3.23	*0.002*
Atrial fibrillation	1.20	0.77–1.88	0.416
Chronic kidney disease	2.83	1.58–5.07	* <0.001*
EuroSCORE II	1.03	0.98–1.08	0.290
Postoperative delirium	1.89	1.06–3.36	*0.030*
Frailty (EFT ≥ 3)	2.06	1.31–3.23	*0.002*
General anesthesia	1.21	0.76–1.93	0.417
Major adverse event	2.03	1.04–3.96	*0.039*

*Italic values seems this highlights the statistically significant values*.

## Discussion

The present study investigated the incidence, predictive factors and its association with survival of POD after TAVR. Main findings of our study are (1) the incidence of POD after TAVR was 10% in this cohort; (2) Patient baseline characteristics including male sex and frailty, as well as peri-procedural complications and general anesthesia emerged as independent predictors of POD; and (3) POD after TAVR was associated with significantly longer ICU- and hospital stay and was a significant predictor of 2-year-mortality, also after adjustment for age, sex, EuroSCORE II, chronic kidney disease, atrial fibrillation, frailty, and the occurrence of major peri-procedural complications.

POD is a frequently observed complication after cardiovascular interventions characterized by an acute onset and fluctuating disturbances of consciousness, attention, cognition, or perception as a result of an insufficient compensation for environmental stressors (1). Thereby, both predisposing factors (e.g., advanced age, frailty, pre-existing cognitive impairment) and precipitating factors (e.g., surgery/intervention, peri-procedural complications) are involved in the development of POD. TAVR patients are frequently elderly, frail and presenting with various comorbidities, thereby exhibiting several predisposing factors for the development of POD. In cardiac surgery, the incidence of POD has been reported to be up to 55% in patients aged ≥ 70-years and is associated with prolonged ICU- and hospital stay and higher mortality (2, 17, 18). In contrast, lower POD rates have been reported after TAVR despite higher age and comorbidity burden, ranging from 7 to 17% (4, 5, 7, 19, 20). Thus, the extent of procedural stress seems to be an important determinant of POD. In line with that, some authors reported higher rates of POD after non-transfemoral TAVR compared to transfemoral TAVR, which could however not be shown in our cohort, most likely due to the low number of patients with alternative access (2.6%) (4, 7, 20).

In our study, atrial fibrillation, male sex, and frailty emerged as significant predisposing factors predicting POD. Atrial fibrillation has been postulated to mediate POD through subclinical cerebral thromboembolic events and hypoperfusion (4, 19, 21). Male sex has been associated with POD in various settings including TAVR, presumably due to a higher prevalence of other predisposing factors (19, 22). The choice of anesthesia (general anesthesia vs. conscious sedation) has been linked to POD in retrospective studies, however in the absence of randomized trials a clear causative relationship remains unproven (23). The decision for general anesthesia may merely be a marker of advanced morbidity and thereby selecting a patient population particularly predisposed to POD due to common risk factors. In general, the identified risk factors are most likely independent from the TAVR procedure and have been reported in other contexts, e.g., orthopedic and general surgery (22, 24).

In addition to baseline characteristics, POD was also associated with a higher rate of peri-procedural complications including stroke, bleeding, infection, and vascular injury. 62.1% of POD cases were associated with a peri-procedural complication. As a consequence, ICU- and hospital stay was significantly longer. As described previously, POD itself was not significantly related with in-hospital mortality after adjustment for those peri-procedural complications (19), indicating that POD alone might not be considered a potentially fatal complication by itself. However, after adjusting for peri-procedural complications and baseline characteristics, POD nevertheless emerged as an independent predictor of 2-year mortality, thus being a risk factor for long-term survival. In addition to higher mortality and prolonged hospital stay, previous studies have reported increased health care costs, sustained cognitive limitation and need for rehabilitation (6, 8, 9, 25). It is difficult to assess to what extent the observed increased morbidity and mortality can be truly attributed to POD itself. Patients developing POD may merely represent a subgroup of patients already predisposed to worse treatment outcomes.

Also, frailty has been described as a predisposing factor of POD in general and as an important determinant of outcome after TAVR (12, 24, 26). Thereby, the predictive value was superior when frailty was assessed with objective tools instead of an informal “eyeball test” (4, 27). The EFT score used in this study has been previously shown to predict 1-year-mortality after TAVR (12). Similarly, an EFT score ≥ 3 significantly predicted POD in our study and was moreover an additional independent predictor of 2-year-mortality. Although frailty and POD seem closely related, both were independently associated with decreased survival.

Taken together, there is increasing evidence associating POD with unfavorable outcomes. Even if POD is not directly linked to mortality, the avoidance of POD seems highly important, especially in the vulnerable TAVR population. Preventive and supportive measures combined with early recognition have been shown to reduce POD and associated health care utilization and hence, might have the potential to improve patient outcome (10). The predictive factors identified in this study can aid in the identification of patients at higher risk for developing POD who may benefit from early recognition and targeted intervention. Although many predisposing and precipitating factors of POD are non-modifiable, several non-pharmacological strategies exist to prevent POD including early mobilization, sleep-wake cycle preservation or cognitive stimulation activities (10, 28). Further studies should evaluate possible preventive strategies and their effect on cognitive and functional outcome.

### Study Limitations

Our study is a single center observational analysis with all inherent limitations. The highly validated CAM-ICU score was used for detection of POD, following current guideline recommendations. However, POD can easily remain unrecognized, especially when hypoactive, thereby underestimating the true incidence of POD. Furthermore, other risk factors, that have been previously associated with POD (e.g., prior silent cerebral ischemic lesions, educational background, sleep disordered breathing, depression), could not be assessed. Finally, there was a relatively high rate of patients lost to follow-up at 2-years.

## Conclusions

POD is common and affecting 10% of patients after TAVR. We identified predisposing factors including male sex, atrial fibrillation, and frailty as well as peri-procedural complications as important predictors of POD. POD was significantly associated with reduced 2-year survival, even after adjustment for baseline characteristics and peri-procedural complications. Consequently, preventive strategies and early recognition may decrease the incidence of POD and improve outcomes.

## Data Availability Statement

The raw data supporting the conclusions of this article are available from the corresponding authors upon reasonable request.

## Ethics Statement

The studies involving human participants were reviewed and approved by University of Cologne, Medical Faculty. Written informed consent for participation was not required for this study in accordance with the national legislation and the institutional requirements.

## Author Contributions

VM, SB, MA, and TR: conception and design. VM, KR, MIK, HW, SL, KE, and EK: data collection. VM, KR, SB, VV, FJ, MA, and TR: analysis and data interpretation. VM, MA, and TR: writing the article. HW, SL, KE, EK, VV, MK, FJ, GN, SB, MA, and TR: critical revision of the article. VM, KR, MIK, HW, SL, KE, EK, SB, MK, GN, VV, FJ, MA, and TR: final approval of the article. All authors contributed to the article and approved the submitted version.

## Conflict of Interest

The authors declare that the research was conducted in the absence of any commercial or financial relationships that could be construed as a potential conflict of interest.
